# Highly improved homopolymer aware nucleotide-protein alignments with 454 data

**DOI:** 10.1186/1471-2105-13-230

**Published:** 2012-09-12

**Authors:** Fredrik Lysholm

**Affiliations:** 1 IFM Bioinformatics and SeRC (Swedish e-Science Research Centre), Linköping University, S-581 83, Linköping, Sweden; 2 Department of Cell and Molecular Biology, Science for Life Laboratory, Karolinska Institutet, S-171 77, Stockholm, Sweden

## Abstract

**Background:**

Roche 454 sequencing is the leading sequencing technology for producing long read high throughput sequence data. Unlike most methods where sequencing errors translate to base uncertainties, 454 sequencing inaccuracies create nucleotide gaps. These gaps are particularly troublesome for translated search tools such as BLASTx where they introduce frame-shifts and result in regions of decreased identity and/or terminated alignments, which affect further analysis.

**Results:**

To address this issue, the Homopolymer Aware Cross Alignment Tool (HAXAT) was developed. HAXAT uses a novel dynamic programming algorithm for solving the optimal local alignment between a 454 nucleotide and a protein sequence by allowing frame-shifts, guided by 454 flowpeak values. The algorithm is an efficient minimal extension of the Smith-Waterman-Gotoh algorithm that easily fits in into other tools. Experiments using HAXAT demonstrate, through the introduction of 454 specific frame-shift penalties, significantly increased accuracy of alignments spanning homopolymer sequence errors. The full effect of the new parameters introduced with this novel alignment model is explored. Experimental results evaluating homopolymer inaccuracy through alignments show a two to five-fold increase in Matthews Correlation Coefficient over previous algorithms, for 454-derived data.

**Conclusions:**

This increased accuracy provided by HAXAT does not only result in improved homologue estimations, but also provides un-interrupted reading-frames, which greatly facilitate further analysis of protein space, for example phylogenetic analysis. The alignment tool is available at http://bioinfo.ifm.liu.se/454tools/haxat.

## Background

In the last decade, next-generation sequencing methods have emerged, become widely used and are today a prerequisite for most large-scale studies. One of the early next-generation techniques is Roche 454 sequencing [1], currently extensively used as it produces longer sequence reads compared to other platforms. The first 454 sequencing platform was introduced in 2005 with the GS20 instrument. The GS20 brought a huge leap in performance over present Sanger based techniques and produced around 20 million bases (Mb) per run [1]. Since then, the 454 technique has repeatedly evolved and the GS FLX Titanium instrument, introduced in 2008, produces as much as 500 Mb per run in reads of approximately 350 bases per read [2]. The long reads produced by 454 sequencing are particularly useful when scaffolding is difficult, where homology to known sequences is distant or absent and overlaps may be ambiguous or non-existent, e.g. *de novo* genome sequencing or metagenomics. The underlying methodology involved in sequence comparisons as well as in most subsequent data processing steps, is sequence alignment, which is an important fundament for the entire field of bioinformatics.

The global sequence alignment problem and a method for solving it was first proposed by Needleman and Wunsch in 1970 [3]. The suggested algorithm utilised a technique called dynamic programming. Dynamic programming solves a problem by dividing the problem into solvable sub-problems. In 1981, Smith and Waterman defined the local alignment and proposed a slightly modified algorithm to solve it [4]. Finally, one year later Gotoh proposed the usage of non-linear gap penalties and the corresponding modified algorithms [5] for solving the global and local alignment problems. While a single sequence alignment is quickly calculated, a full database search using Smith-Waterman-Gotoh would be unnecessarily time consuming as most comparisons would render non-significant alignments. To address this issue, heuristic methods such as FASTA [6,7] and BLAST [8,9], which greatly reduces the number of sub-problems solved, were developed. Later, methods using improved heuristics, and/or innovative implementations to more efficiently utilise computer memory and processors have been proposed, e.g. MegaBLAST [10], SSAHA2 [11], BLAT [12] and Smith-Waterman-Gotoh alignments utilising SIMD instructions [13].

The characteristics of 454 data are to some extent different from most other sequencing technologies. 454 sequencing is a pyrosequencing method where the nucleotide reagents for detecting thymine (T), adenine (A), cytosine (C) and guanine (G) are repeatedly cycled over each DNA template fragment, while elongating the complementary strand. The intensity of each flow of nucleotide reagents is recorded, as a so-called flowpeak and collected in a flowgram [1]. As the homopolymer length is estimated from peak intensity, occasional uncertainties occur [1]. These uncertainties are challenging for downstream analysis algorithms. Current analysis methods do not provide any modification to the alignment algorithm, but instead recommend using adjusted heuristics, e.g. a shorter indexing word length, to cope with this problem. While a majority of the homopolymer insertions/deletions (indels) are placed correctly by gaps, close to the end of the read, the alignments are often truncated and sequential undercall-overcall (and vice versa) are often mistaken for a single nucleotide polymorphism (SNP). The first method that addressed the problem of homopolymer uncertainties was FLAT [14], which performs probabilistic flowgram matching. The downside of using flowgram matching is the limited possibility to detect non-identical matches, apart from homopolymer dissimilarities. Another tool that addressed this problem was PanGEA [15], which uses a modified Smith-Waterman-Gotoh alignment algorithm, which allows gaps at the end of long homopolymer runs at a lowered cost. FAAST [16] was developed in order to combine the benefits of both methods. FAAST utilises the flowgram to dynamically set a position specific homopolymer gap penalty relative to the uncertainty at that position, regardless of homopolymer length.

Since nucleotide alignments fail to produce significant results for remote homologues, homopolymer indel recover methods based on nucleotide alignments are not applicable at remote homology. Due to this limitation of nucleotide alignments, many metagenomic studies [17-19] perform translated homology searches (against a protein database), using translated BLAST, e.g. tBLASTx or BLASTx [9]. However, BLAST does not handle frame-shifts and at best presents two alignments in each frame, and these may be either significantly separated or overlapping, depending on the similarity in the shifted frame. These issues add complexity to metagenomic analysis, and potential homopolymer inaccuracies often have to be corrected manually to preserve an open reading frame (ORF). There are methods that do allow nucleotide-protein alignments with frame-shifts, for example the method proposed by States and Botstein [20]. However, none of these methods take homopolymer errors into account, which makes them unsuitable for 454 data or other data where homopolymer reading errors occur, for example the Ion Torrent platform [21].

To address these problems, I here introduce a new tool named HAXAT (Homopolymer Aware Cross Alignment Tool). This new tool solves the protein-nucleotide alignment through a novel dynamic programming algorithm. The algorithm is based on the Smith-Waterman-Gotoh algorithm, and is capable of handling frame-shifting matches. It considers homopolymer inaccuracies through position-specific penalties, provided by a pre-calculated query profile. Through this novel algorithm, HAXAT produces more sensitive results with 454 data, even in the absence of flowpeak information (FASTA input).

## Results

The new tool HAXAT addresses the issues of frame-shifts in translated nucleotide – protein alignments while considering an underlying 454 data model. HAXAT utilises a novel alignment algorithm, with corresponding parameters, to perform these alignments. HAXAT was designed to be useful in the analysis of metagenomic data, but it can be used in various genome sequencing and annotation tasks, such as ORF annotation.

### The alignment output format

**Figure 1:**
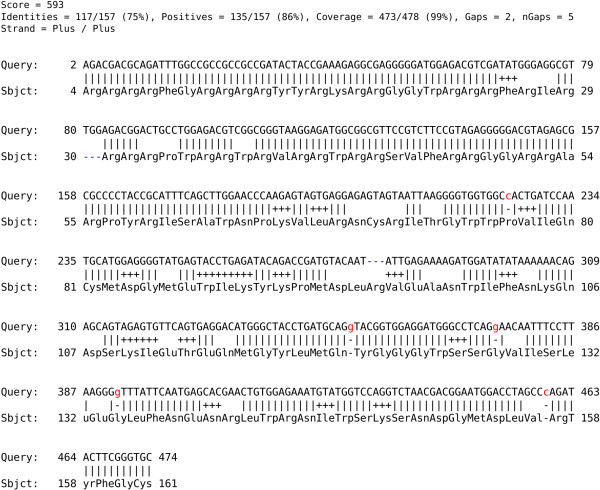
**Alignment of a read generated from GU570202 against ADD46842.** The figure illustrates the HAXAT alignment of a Titanium read generated from ORF1 of GU570202 aligned against ADD46842. Five frame-shift corrections were found and are highlighted in red and with lower case characters. The first correction, the “c”, is aligned against a corresponding database position and is therefore regarded as an inserted nucleotide (predicted undercall). The next consequent correction, the first “g” is aligned against a database gap and is thus a deleted nucleotide (predicted overcall). The alignment is produced using BLOSUM62, a gap open/ext cost of 8/2 as well as a single/double nucleotide gap cost of 16/28 and the homopolymer parameters “h = 0.5 and t = 4” (default parameters).

HAXAT produces its output in a new alignment format to incorporate the protein-nucleotide alignments as well as annotations for the frame-shifting positions. An alignment of a simulated 454 Titanium read from ORF1 of Torque Teno Sus Virus 1, GU570202 against ADD46842 is shown in Figure 1. All frame-shifting insertions/deletions (indels) are marked in the alignment output by lowercase characters. Furthermore, for each nucleotide gap caused by a frame-shift, an inserted nucleotide is predicted based on a maximum likelihood estimate (the nucleotide and penalty combination that maximises the score). Apart from reporting the score, identities, positives, coverage and gaps, the output format also specifies the number of frame-shifting gaps (nucleotide space gaps, see “nGaps” in Figure 1).

### Evaluation of homopolymer insertion/deletion recovery

A representative benchmark set with different degrees of difficulty was constructed from a single coding seed sequence. The ORF1 of Torque Teno Sus Virus 1, GU570202, was chosen as seed sequence. A Torque Teno virus sequence represents a likely single read sequence, resulting from a metagenomics or genome project, as TT viruses are highly heterogeneous [22]. The nucleotide sequence was first translated into its corresponding amino acid sequence. Six additional sequences, one for each degree of difficulty, were subsequently constructed through mutations in protein space down to 90, 80, 70, 60, 50 and 40% identity. Each mutation position was randomly chosen, while the substitution frequencies were proportional to the corresponding BLOSUM(*X*) substitution probabilities [23], where *X* denotes the clustering identity threshold used. In order to supply query sequences, 1,000 454 Titanium reads were simulated by the “454sim” simulator [24], using default settings. Finally, 4216 parameter combinations were evaluated for all seven degrees of difficulty, by alignment of the 1,000 reads to each of the seven target protein sequences. The complete study design and evaluation scripts are available in Additional file 1. HAXAT was executed using the BLOSUM62 substitution matrix and a gap open/ext cost of 8 and 2 (default settings).

### Parameter evaluation setup

The predicted homopolymer inaccuracies were extracted from the alignment and the prediction accuracy was evaluated. A correct prediction was defined as either a correctly predicted deletion (flowpeak overcall) or correctly placed insertion (flowpeak undercall) of a particular nucleotide. Consequently, an incorrect prediction is an incorrectly placed deletion/insertion or an insertion of an incorrect nucleotide at a particular position. Homopolymer indels not covered by the alignment were not regarded as a false negative prediction (results where these are counted as *FN* are available in Additional file 1, showing highly similar results). The parameters evaluated were; the cost for opening a single (−*S* parameter) or a double (−*D*) frame-shifting gap. The 454 model specific parameters evaluated were; the homopolymer frame-shifting gap penalty fraction (−*h*) which determines the minimum single and double frame-shifting penalties (*Sh* = *S · h* and *Dh* = *D · h*), the relative flowpeak deviation allowed (−*k*) and whether to use insertion validation or not (−*V*), see Methods. HAXAT can be executed in three different modes by applying two different alignment models. The first mode uses a neutral (non-454-aware) alignment model (activated by the *-F0* parameter with the HAXAT tool). Consequently, for this model, only a single (−*S*) and double frame-shifting gap cost (−*D*) is applicable. This model is highly similar to the method previously proposed by States and Botstein [20], while it provides the possibility to set different costs to single and double gaps. The second mode uses a 454 aware alignment model without flowpeak information, i.e. running HAXAT with FASTA input (e.g. homopolymer aware alignment). Finally, the last mode applies the same 454 aware alignment model but uses flowpeak information through SFF input (e.g. flow-space aware alignment). The prediction accuracy was evaluated by the Matthews Correlation Coefficient (*MCC*) [25] for all three modes.

### Evaluation of parameters for a neutral alignment model

**Figure 2:**
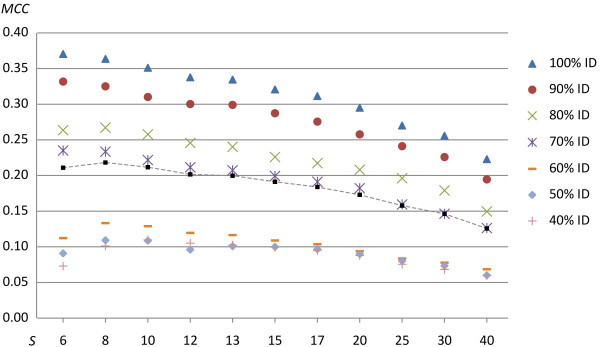
**Single gap penalty evaluation for a neutral (non-454 aware) model.** The figure describes the alignment accuracy through Matthews Correlation Coefficient (y-axis) of the homopolymer insertion/deletion recovery versus the single gap penalty (x-axis). The accuracy is given for each of the seven degrees of difficulty, i.e. alignments against targets of an identity ranging from 100% down to 40% identity. Furthermore, the mean accuracy for all seven degrees of difficulty, at each gap penalty, is also shown by squares and a dashed line.

A total of 72 parameter combinations employing the neutral alignment model were evaluated (−*F0*), for each of the seven degrees of difficulty (targets ranging from 100% down to 40% identity). From that set, the maximum prediction accuracy (*MCC*-value) for each tested value of the single gap penalty was extracted, see Figure 2. The results showed that the accuracy peaked at the smallest allowed single gap penalty (6) for high identity targets, while at lower identity the accuracy peaked at a slightly higher penalty (8 and 10). The mean accuracy of all targets peaked at 8. The double gap penalty was evaluated at 1.4 to 2 times the single gap penalty, but the experiments showed a peak accuracy for a double gap penalty that was 1.9 or 2 times the single gap penalty. However, due to the rarity of double homopolymer under/overestimates, the double gap penalty does not affect the results significantly in these evaluations, see Additional file 2: Figure S1. Table 1 describes the prediction accuracy obtained for the highest scoring parameter combinations of each target.

**Table 1:** Parameter evaluation using a non-454 model

**Parameters/ID**	**40%**	**50%**	**60%**	**70%**	**80%**	**90%**	**100%**
*S = 10, D = 20, F = 0*	**0.1098**	0.1064	0.1259	0.2216	0.2566	0.3099	0.3512
*S = 8, D = 14, F = 0*	0.1006	**0.1092**	0.1310	0.2332	0.2659	0.3245	0.3632
*S = 8, D = 12, F = 0*	0.0976	0.1074	**0.1333**	0.2323	**0.2672**	0.3232	0.3619
*S = 6, D = 10, F = 0*	0.0703	0.0834	0.1087	**0.2350**	0.2613	0.3292	0.3684
*S = 6, D = 12, F = 0*	0.0731	0.0909	0.1122	0.2346	0.2635	**0.3317**	**0.3707**
***S = 8, D = 15, F = 0***	0.1012	0.1070	0.1311	0.2332	0.2664	0.3251	0.3638

The table describes the Matthews Correlation Coefficient of the homopolymer insertion/deletion recovery for various gap costs using a non-454 data model. The results are evaluated using alignments against database entries of a protein identity ranging from 40 to 100%, not including potential sequencing errors. The best results are highlighted in bold for each column. The parameter set which results in the best mean *MCC* is highlighted in bold.

### Evaluation of parameters for homopolymer aware alignments

**Figure 3:**
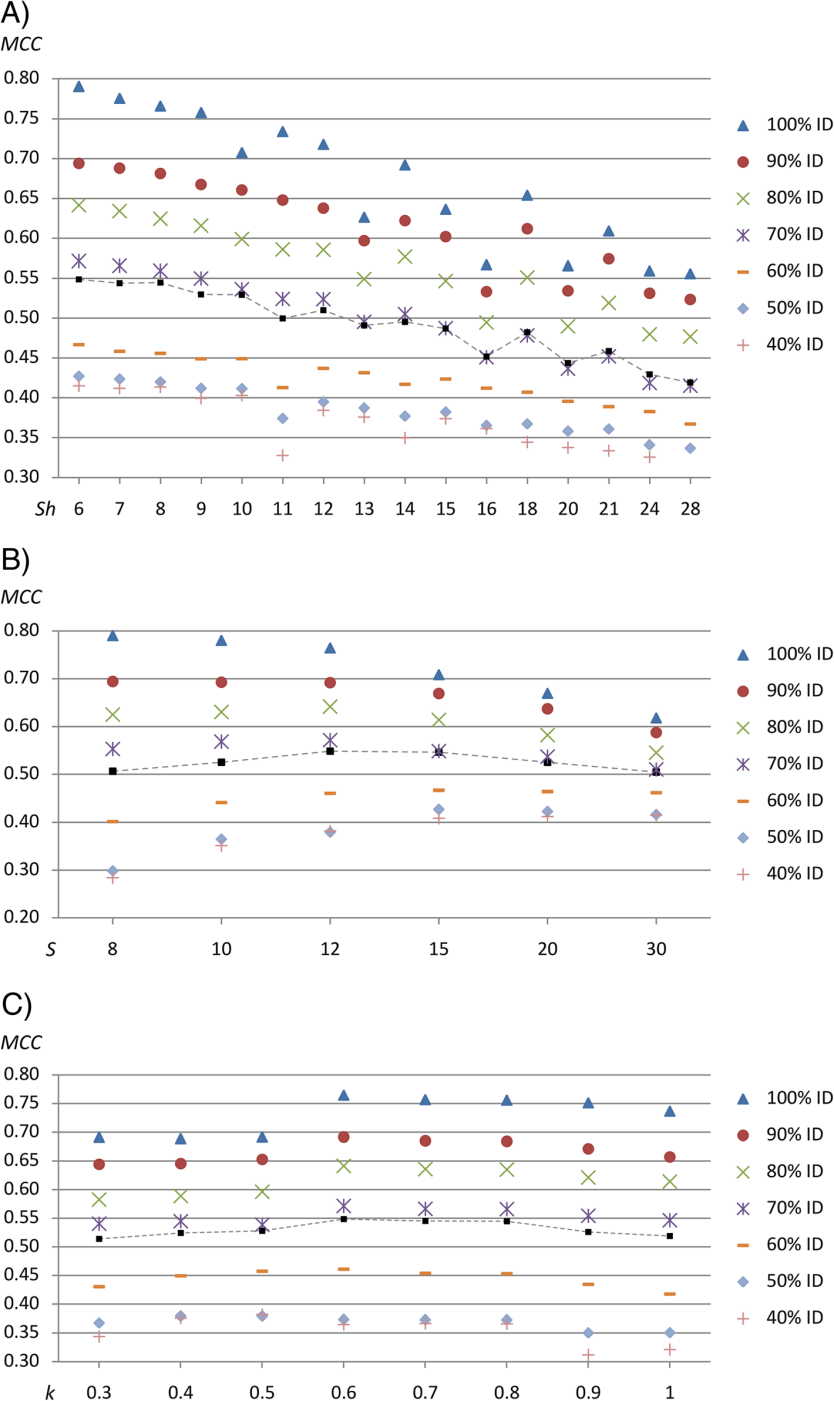
**Parameter evaluation for a 454-aware model, without flowpeak information.** The figure describes the single homopolymer gap penalty (*Sh = S · h*) in Panel **A**, the single gap penalty (*S*) in Panel **B**, and the flowpeak deviation (*k*) in Panel **C**. The parameter values are evaluated by Matthews Correlation Coefficient (y-axis) of the homopolymer insertion/deletion recovery and each parameter value are given on the x-axis. Each figure plots the values obtained for all seven targets ranging from 100% down to 40% identity. In addition, the mean accuracy is also shown by squares and dashed lines.

A total of 4144 parameter combinations for each of the seven degrees of difficulty (targets ranging from 100% down to 40% identity) were evaluated, see Figure 3. As seen in Figure 3A, the *h*-value was chosen so that the homopolymer single gap penalty (*Sh = S · h*) assumed the lowest allowed value. As the parameter combinations evaluated did not span all possible values of all parameters, some combinations received lower *MCC*-values than expected. The single gap penalty, Figure 3B, assumed different peak values for different target identities. For low identity targets a high single gap penalty was favoured and at high identity targets a low single gap penalty was favoured. The single gap penalty determined the cost of introducing a frame-shifting gap in regions where long homopolymers are not present. At high identity, it was favourable to introduce gaps to keep high identity while at low identity, the risk of introducing invalid gaps were higher and thus better results were produced with an increased gap cost. Finally, Figure 3C, shows the flowpeak deviation parameter, *k*. As with the single gap penalty, high identity targets allowed a more aggressive penalty decrement at increased homopolymer length compared to low identity targets. For example, at 100% identity, the highest value evaluated, *1.0*, produces the peak accuracy while at the mean identity, the peak was at *k = 0.6*.

As with the neutral (non-454-aware) model, the double/single gap penalty ratio was evaluated from 1.4 to 2.0 where values around 2.0 were found to be favoured slightly, see Additional file 3: Figure S2. Table 2 describes the prediction accuracy obtained for the highest scoring parameter combinations of each target. We note from the table that flow-order insertion validation (−*V*) generally performs worse. A substantial increase in accuracy was achieved using a homopolymer aware model, where for instance the *MCC*-value at 100% rose sharply to 0.791, while at 40% a *MCC*-value of 0.415 was obtained, compared to 0.371 and 0.110 for the neutral model.

**Table 2:** Parameter evaluation using a 454-model without flowpeak information

**Parameters/ID**	**40%**	**50%**	**60%**	**70%**	**80%**	**90%**	**100%**
*S = 30, D = 54, h = 0.2, k = 0.9, V = 1*	**0.415**	0.415	0.462	0.493	0.538	0.571	0.605
*S = 15, D = 26, h = 0.4, k = 0.6, V = 0*	0.402	**0.427**	0.464	0.547	0.606	0.665	0.706
*S = 15, D = 29, h = 0.4, k = 0.6, V = 0*	0.403	0.425	**0.467**	0.548	0.607	0.666	0.708
***S = 12, D = 24, h = 0.5, k = 0.6, V = 0***	0.351	0.359	0.461	0.571	0.640	0.691	0.765
*S = 8, D = 15, h = 0.7, k = 1, V = 0*	0.259	0.261	0.363	0.553	0.625	**0.694**	**0.791**

The table describes the Matthews Correlation Coefficient of the homopolymer insertion/deletion recovery for various parameter combinations using a 454 model without flowpeak information (i.e. FASTA input). The results are evaluated using alignments against targets of a protein identity ranging from 40 to 100%, not including potential sequencing errors. The best results are highlighted in bold for each column as well as the parameter set which results in the best mean *MCC*.

### Evaluation of parameters for a flowpeak aware alignment model

**Figure 4:**
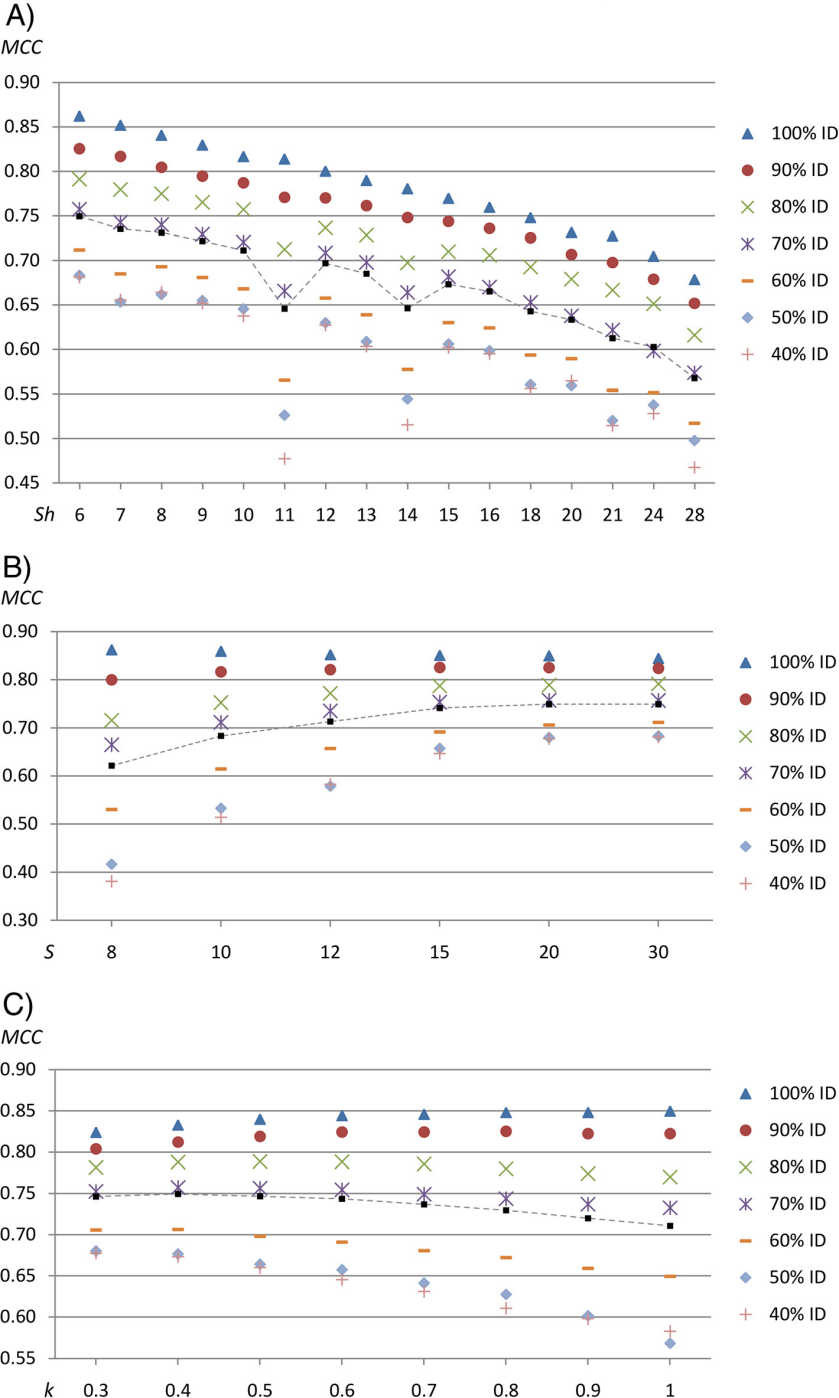
**Parameter evaluation for a 454-aware model, using flowpeak information.** The figure describes the single homopolymer gap penalty (*Sh = S · h*) in Panel **A**, the single gap penalty (*S*) in Panel **B**, and the flowpeak deviation (*k*) in Panel **C**. The parameter values are evaluated by Matthews Correlation Coefficient (y-axis) of the homopolymer insertion/deletion recovery and each parameter value are given on the x-axis. Each figure plots the values obtained for all seven targets ranging from 100% down to 40% identity. In addition, the mean accuracy is also shown by squares and dashed lines.

The final parameter evaluation was performed using the same 454-model but by using flowpeak information through feeding HAXAT with raw 454 data input (i.e. SFF-file input), see Figure 4. Just as without flowpeak information a low homopolymer single gap penalty was found to be favoured, Figure 4A, while in general due to more complete flowpeak information a higher single gap penalty was preferred, see Figure 4B. Figure 4C shows the flowpeak deviation parameter, *k*. At low identity, a lower value of *k* (a more restrictive penalty drop-off) was found to be optimal, while at high identity, large values of *k* produced the best results. The double/single gap penalty ratio was again evaluated from 1.4 to 2.0 and values around 2.0 were slightly more favourable, see Additional file 4: Figure S3. Finally, Table 3 describes the prediction accuracy obtained for the highest scoring parameter combinations of each target. It is noteworthy that using flow-order insertion validation (−*V*) produced better results for all cases except at 100% target identity. The increase in accuracy, compared to using no flowpeak information, was most notable when the target identity was low, due to the absence of biological information (low identity). For example, at 40% identity with flowpeak information, a *MCC*-value of 0.68 was obtained, compared to 0.41 without the aid of flowpeak information. For a complete list of the parameters examined, for all three evaluations, and a list of all true/false positives/negatives, see Additional file 1.

**Table 3:** Parameter evaluation using a 454-model using flowpeak information

**Parameters/ID**	**40%**	**50%**	**60%**	**70%**	**80%**	**90%**	**100%**
*S = 30, D = 60, h = 0.2, k = 0.4, V = 1*	**0.681**	0.682	0.708	0.748	0.775	0.796	0.815
*S = 30, D = 60, h = 0.2, k = 0.5, V = 1*	0.681	**0.683**	**0.712**	0.752	0.782	0.803	0.823
***S = 20, D = 40, h = 0.3, k = 0.4, V = 1***	0.673	0.677	0.706	**0.757**	0.788	0.812	0.833
*S = 30, D = 60, h = 0.2, k = 0.8, V = 1*	0.666	0.671	0.701	0.757	**0.791**	0.817	0.839
*S = 15, D = 30, h = 0.4, k = 0.5, V = 1*	0.613	0.629	0.673	0.744	0.782	**0.825**	0.849
*S = 8, D = 15, h = 0.7, k = 0.4, V = 0*	0.321	0.317	0.455	0.639	0.697	0.774	**0.862**

The table describes the Matthews Correlation Coefficient of the homopolymer insertion/deletion recovery for various parameter combinations using flowpeak information (i.e. SFF input). The results are evaluated using alignments against targets of a protein identity ranging from 40 to 100%, not including potential sequencing errors. The best results are highlighted in bold for each column as well as the parameter set which results in the best mean *MCC* (default parameters).

In conclusion, due to the low number of homopolymer indels (positives) in comparison to non-mutated positions (negatives) even with only a few misplaced indels, each resulting in a consequent FP and FN, it is difficult to reach a high *MCC*-value. This is clear from Table 3 where, even at 100% protein identity and using flowpeak information, the best *MCC*-value was 0.862. When examined, many of the indels were placed just a few bases away or even at the same location but with the adjacent homopolymer corrected, for example “TTTtAA” instead of the correct “TTTaAA”. As seen in the Tables 1, 2 and 3, in all cases, the novel 454 model significantly outperformed the previously existing neutral (non-454-aware) alignment model, regardless of the parameter settings.

### Effects of using the HAXAT alignment algorithm on real data

**Figure 5:**
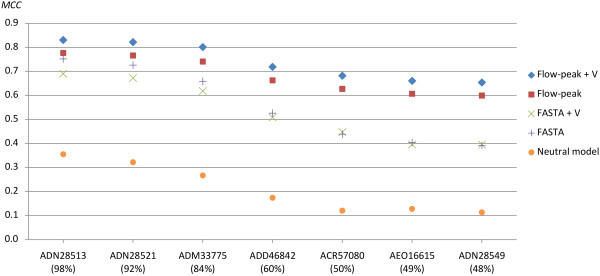
**Alignment accuracy results for the different models.** The figure shows the alignment accuracy through Matthews Correlation Coefficient (y-axis) of the homopolymer insertion/deletion recovery, for targets and various alignment models. The targets (x-axis) are given by Genbank identifier with the BLAST local identity in parenthesis. The models evaluated are; a 454 model using flowpeak information with and without validation (denoted + V) as well as without flowpeak information (FASTA input) with and without validation and finally a neutral (non-454-aware) model (FASTA input). The alignments were performed using HAXAT and default parameters for a 454 model (with flowpeak information: *-S20 -D40 -h0.3 -k0.4*, without flowpeak information: *-S12 -D24 -h0.5 -k0.6*) as well as the optimal parameters for the neutral (non-454-aware) model on 454-data, *-S8 -D15*.

To further test the alignment models, alignments were performed against real sequences homologues to GU570202 (the seed sequence). The database sequences used are described in Table 4. The alignments were performed using a neutral (non-454) model and a 454-model with/without insertion validation (see Methods), and with/without flowpeak information. As with previous tests, a 1,000 reads were simulated from each homologue and homopolymer errors were estimated from the resulting alignments towards the seed sequence. True/false positives/negatives were defined as before and the algorithm efficiency were evaluated by *MCC*, see Figure 5. The alignments were performed using HAXAT and default parameters for a 454 model (with flowpeak information: *-S20 -D40 -h0.3 -k0.4*, without flowpeak information: *-S12 -D24 -h0.5 -k0.6*) as well as the optimal parameters for the neutral (non-454-aware) model on 454-data, *-S8 -D15* (bold parameter sets in Table 1, 2, and 3). The results from this test, largely, agree with the tests using simulated data. Consistently for all targets, a 0.5 to 0.6 *MCC*-value gap was found between the previously available neutral model and the 454 aware model using flowpeak information and insertion validation. A slight increase in accuracy was achieved with insertion validation when using flowpeak information, while a slight decrease in accuracy was found when flowpeak information was unavailable.

**Table 4:** Sequences homologues to GU570202

**Entry ID**	**Identity**	**Positives**	**Coverage**	**Gaps**	**Score**
*ADN28513.1*	576/589 (98%)	580/589 (98%)	589/648 (91%)	0	1125 bits
*ADN28521.1*	544/589 (92%)	565/589 (96%)	589/648 (91%)	0	1076 bits
*ADM33775.1*	497/590 (84%)	533/590 (90%)	590/648 (91%)	1	984 bits
*ADD46842.1*	355/590 (60%)	442/590 (75%)	590/648 (91%)	14	737 bits
*ACR57080.1*	258/521 (50%)	348/521 (67%)	521/648 (80%)	15	543 bits
*AEO16615.1*	249/513 (49%)	328/513 (64%)	513/648 (79%)	15	506 bits
*ADN28549.1*	288/595 (48%)	393/595 (66%)	595/648 (92%)	17	552 bits

The table shows the BLAST statistics for some homologues to GU570202. The BLASTx program was used with default parameters against with the nucleotide GU570202 ORF1 sequence as query. The identities given are averages for the aligned region while partial alignments relevant for the alignment of a read may obtain both a lower and higher identity.

### Implementation and availability

The homopolymer aware cross alignment algorithm has been implemented in a tool called HAXAT (Homopolymer Aware X-Alignment Tool). HAXAT was implemented in C++ and it compiles using GNU GCC or Intel ICC on both Linux/Windows. The tool is open source under the GNU GPL licence and available as source code and pre-compiled binaries at http://www.bioinfo.ifm.liu.se/454tools/haxat. HAXAT is able to use a wide range of parameters for adjusting the alignment model. More information about parameters, usage and input/output formats can be found in the documentation at the webpage. The webpage also provides a web-version of HAXAT which can either align two sequences using HAXAT alone or search a query sequence against a database using BLASTx heuristics.

## Discussion

As shown above, HAXAT introduces a novel sensitive tool for performing frame-shifting nucleotide-protein alignments using dynamic programming. The increased sensitivity stems from retaining flowpeak information and applying a 454 model to the frame-shifting gap-penalties. HAXAT is intended as an alignment refinement tool in down-stream studies of sequences suspected to contain homopolymer indels. At this moment, HAXAT does not implement any heuristics and performs full dynamic programming calculations for each query-database combination. This greatly reduces the speed at which a full database scan can be performed using this tool alone. On the other hand, a full alignment ensures that the optimal local alignment, given the alignment model, is found. Furthermore, many tools implement excellent heuristics, for example alignment search tools like TFASTA [7], BLASTx [9], and these can be used to reduce the number of alignments computed by HAXAT. An example on how to achieve HAXAT results with the aid of BLASTx heuristics (BLAST + package), using four simple commands, is available at the webpage (http://www.bioinfo.ifm.liu.se/454tools/haxat). The webpage also provides the possibility to run HAXAT directly, both for alignment of two sequences and running a query against a database using BLAST heuristics. Even though HAXAT is developed to handle 454 data, the algorithm could also be applied to other pyrosequencing methods or any sequencing method or data where homopolymer inaccuracies occur, for example the Ion Torrent platform [21].

The HAXAT alignment algorithm is optimised to isolate the most costly calculations so that these can be performed beforehand and stored into a query profile. The calculations of each cell introduces four additional evaluations and references at a maximum *i-1, j-5*, see Methods, in comparison to the Smith-Waterman-Gotoh algorithm which references *i-1, j-1*. This implementation is computationally efficient, but it has some limitations. Most notably; no homopolymer correction tracking is employed and thus it is possible to correct a homopolymer position several times. For example, if the single frame-shifting gap penalty is smaller than half of the double gap penalty, two single gaps would be favoured in cases where the double gap is between two codons (thus interchangeable with two single gaps). Furthermore, the single gap penalty plus the double gap penalty should be larger than the penalty for introducing a gap in protein space, to ensure that protein gaps are not substituted with homopolymer corrections. The HAXAT tool solves these issues by not allowing such penalty combinations. While this limited model for correcting homopolymer indels cannot stably correct homopolymer lengths at low costs, the results show that this model still provides a significant step forward in performance compared to the previously available neutral (non 454-aware) model.

The HAXAT algorithm has been put to the test, both in alignments against simulated data where the identity is fairly uniform (does not vary much along the sequence) and in alignments with real homologues containing low identity regions and complicating gaps. The algorithm provides a substantial gain compared to the previously available neutral model. Additionally, the ability to place homopolymer indels correctly does not only increase accuracy but also the query coverage (see Additional file 1) in these alignments as fewer alignments are terminated by the frame-shift.

## Conclusions

Sequencing methods based on sequencing by synthesis without a terminator, for example 454 sequencing, suffer from frequent homopolymer indels. As most previous sequencing techniques do not show this type of error, many bioinformatics algorithms are not well adapted to handle homopolymer indels. This paper presents a novel algorithm for solving the fundamental problem of sequencing alignment between a nucleotide sequence, potentially burdened by homopolymer reading errors, and a protein sequence. This algorithm has been implemented in an open source tool called HAXAT, available as source code, pre-compiled binaries and through a web-based tool. The results show that HAXAT provides a significant improvement in alignment accuracy for this type of data. The improved accuracy stems from the homopolymer aware algorithm which makes use of raw flowpeak values to further improve homopolymer length predictions. HAXAT provides an important step forward in solving the ‘homopolymer problem’ which faces bioinformaticians when working with 454 data. HAXAT will be useful in metagenomic and genomic analysis, not only for 454 sequencing data, but also for emerging methods, such as the Ion Torrent platform. The HAXAT tool is open source under the GNU GPL licence and is publically available as source code, pre-compiled binaries and as a web-based tool at http://www.bioinfo.ifm.liu.se/454tools/haxat.

## Methods

### Dynamic programming alignment algorithm

The novel alignment algorithm is a Smith-Waterman-Gotoh inspired dynamic programming algorithm and aligns a nucleotide sequence (horizontal) with a protein sequence (vertical). The dynamic programming table is defined with a cell for each protein and nucleotide sequence position, thus examining all three forward frames. As a consequence, the algorithm is modified for the match and protein gaps so that each step in nucleotide sequence space is in jumps of 3 instead of 1. Additionally, the nucleotide sequence can be gapped in a frame-breaking fashion through matching 1, 2, 4 and 5 nucleotides to one amino acid. This corresponds to; insertion of 2 nucleotides, 1 nucleotide, deletion of 1 nucleotide and 2 nucleotides, respectively.

More formally, the local nucleotide-protein alignment dynamic programming cell score is calculated according to Equation 1.

(1) $$S_{i,j}=max\{\begin{array}{c} \begin{array}{l} S_{i-1,j-1}+m_{1}(p_{i,}q_{j})\\ S_{i-1,j-2}+m_{2}(p_{i,}q_{j-\text{1.}j})\\ S_{i-1,j-3}+m_{3}(p_{i,}q_{j-\text{2.}j})\\ S_{i-1,j-4}+m_{4}(p_{i,}q_{j-\text{3.}j})\\ S_{i-1,j-5}+m_{5}(p_{i,}q_{j-\text{4.}j}) \end{array}\\ E_{i,j}\\ H_{i,j}\\ 0 \end{array}$$

The variable *S*_*i,j*_ represents the optimal cell score in position *i,j*. The protein sequence is represented by *p* and thus *q* is the nucleotide query sequence. *p*_*i*_ is an amino acid in *p* at position *i* and *q*_*j*_ is the nucleotide *j* in *q* (*q*_*j-n.j*_ denotes a range of *n + 1* nucleotides that ends in *j*). The function, *m*_*3*_*(p*_*i*_*, q*_*j-2.j*_*)*, determines the score of aligning the amino acid *p*_*i*_ with the *q*_*j-2.j*_ nucleotide triplet. In general, the score of aligning *n* nucleotides with the amino acid *p*_*i*_ determined by the *m*_*n*_*(p*_*i*_*, q*_*j-n+1.j*_*)*, where *n = 3* is the trivial case of aligning a complete codon to an amino acid. Finally, *E*_*i,j*_ and *H*_*i,j*_ describe the optimal cell score that ends with a gap in the sequence *p* and *q*, respectively. The variables are calculated through; *E*_*i, j*_ *= max(S*_*i-1, j*_*- G*_*0*_*, E*_*i-1, j*_*- G*_*e*_*)* and *H*_*i, j*_ *= max(S*_*i, j-3*_*- G*_*0*_*, H*_*i, j-3*_*- G*_*e*_*)*, where *G*_*0*_ is the minimum gap penalty and *G*_*e*_ is the gap extension penalty.

### Defining the frame-breaking match function

The frame-breaking match function defined for matching 1, 2 and 4, 5 nucleotides to an amino acid can be considered as inserting 2 and 1 as well as deleting 1 and 2 nucleotides in order to get a nucleotide triplet. The resulting match score is thus a composite score of the gap penalty and the match score of the resulting nucleotide triplet against the amino acid.

**Figure 6:**
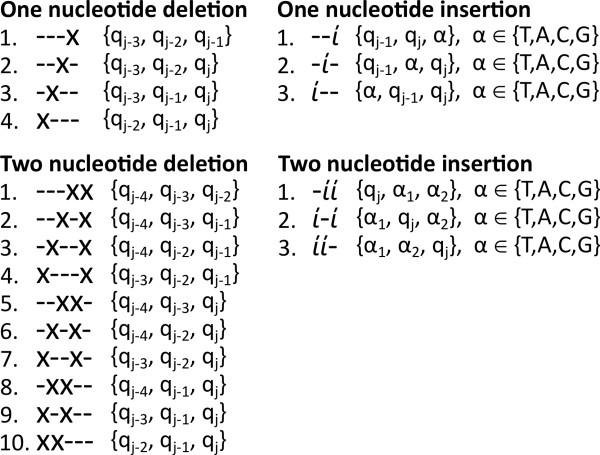
**Frame-shifting deletion/insertion possibilities.** The figure describes the frame-shifting deletion/insertion possibilities as well as which nucleotide query positions are kept, for the four deletion/insertion events. There are 4 possible ways of deleting a nucleotide amongst 4 nucleotides and 10 possible combinations of deleting two nucleotides amongst 5. There are 3 possible ways of inserting one and two nucleotides. However, given that the inserted nucleotide α ∈ {T,A,C,G}, there 10 non-overlapping triplets (4 + 3 + 3) for the single insert and 37 (4^2^ + 4 x 3 + 3^2^) for the double insert.

A deletion match is obtained by deleting one or two nucleotides to retain a nucleotide triplet. In the case of four nucleotides, there are 4 possible deletions which would render a triplet (*4 choose 1 = 4*). Given five nucleotides, a double deletion could occur in 10 unique ways (*5 choose 2 = 10*), see Figure 6. Similarly, there are three possible ways to insert both one and two nucleotides to form a triplet (*3 choose 1 = 3 choose 2 = 3*). However, given that the inserted nucleotide α ∈ {T,A,C,G} there are 12 (4 × 3) combinations to insert one nucleotide, and 48 (4^2^ × 3) combinations to insert two nucleotides, some of which overlap. In fact, for one insert there are 10 non-overlapping triplets (4 + 3 + 3) and for two inserts 37 (4^2^ + 4 × 3 + 3^2^) non-overlapping triplets. The _*mn*_*(p*_*i*_*, q*_*j-n+1.j*_*)* function can be defined according to Equation 2.

(2) $$M_{n}\left(p_{i,}q_{j-n+\text{1.}j}\right)=max_{k}\left(m_{3}\left(p_{i,}T_{k}\right)-P_{k}\right)$$

The variable *T*_*k*_ represents one of the *k* possible nucleotide triplets, see Figure 6, and *P*_*k*_ the insertion/deletion penalty. Finally, the *m*_*3*_*(p*_*i*_*, T*_*k*_*)* function is simply the substitution score given by any substitution matrix where the *T*_*k*_ triplet is translated to the corresponding amino acid.

### Position specific frame-shift penalty

The position specific frame-shift penalty is divided into a single and a double gap penalty. The single insertion/deletion match path employ the single gap penalty while the double insertion/deletion match path apply the double gap penalty for the grouped deletes/inserts and the double single gap penalty (2 × Single gap penalty) for a non-grouped deletes/inserts, see Figure 6 for delete/insert possibilities.

Apart from the basic frame-shift penalty, a penalty at reduced cost is allowed at the end of each homopolymer stretch. The homopolymer single/double gap penalty is smaller than the regular single/double gap penalty, *P*_*0*_, and decreases with an increased flowpeak uncertainty up to *P*_*h*_ *= P*_*0*_*· h*. Thus, the penalty decrement can be described as *P*_*ħ*_*· f*, where *f* denotes the factor by which the penalty is reduced, and *P*_*ħ*_ *= P*_*0*_*· (1 - h)*. The penalty in turn is proportional to the relative flowpeak deviation required for an *n*-mer to be called as an *m*-mer and scaled by 1/*k*, see Equation 3.

(3) $$\begin{array}{l} f=1-\left(\frac{1}{k}\cdot \frac{Dev_{m}\left(p,n\right)}{\max\left(n,1\right)}\right),\;Dev_{m}\\ \;=\left\{\begin{array}{c} p-m-0.5\\ 0\\ m-0.5-p \end{array},\;,\begin{array}{c} m<n\\ m=n\\ m>n \end{array}\right) \end{array}$$

**Figure 7:**
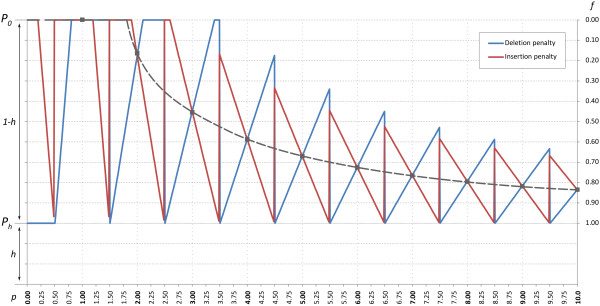
**Frame-shifting penalty function.** The figure shows the insertion and deletion penalty, *P = P*_*0*_*- P*_*ħ*_*· f, P*_*ħ*_ *= P*_*0*_*· (1-h),* on the left y-axis as well as the gap penalty reduction factor, *f*, on the right y-axis for flowpeak values between 0 and 10 (x-axis). The penalties intersect at each integer peak value, noted by squares and the dotted line shows the 1/*n* slope of the *f*-function. The homopolymer length in this plot is assumed to be the integer value of the flowpeak value *p* (x-axis), thus *p* satisfies; *n-0.5 ≥ p > n + 0.5*, except for deletion penalties for *p < 0.5* where a 1-mer is assumed.

Consequently, the *k* parameter describes at which relative flowpeak deviation the full gap penalty is used. For instance, if *k = 0.2* is used; the gap penalty at an 1-mer peak deviating less than 0.2 or a 2-mer peak deviating less than 0.4 will be reduced, and at 0.2 and 0.4 and above the full penalty will be employed, respectively. For non-454 data the flowpeak value, *p*, is equal to the homopolymer length, *n*, and thus the up-calling (*Dev*_*n+1*_*(n, n)*; insert) and down-calling (*Dev*_*n-1*_*(n, n)*; delete) deviations are equally large, and consequently so are *f* and the insertion/deletion penalty, see Figure 7. However, in the more general description the flowpeak value, *p*, may be any floating point value (although assumed to satisfies *n-0.5 ≥ p > n + 0.5*). If the flowpeak value is not equal to the homopolymer length the insertion/deletion penalty thus differ, see Figure 7, Equation 3. For example, given a flowpeak value of 1.50, thus called as a 2-mer, the deletion penalty is much smaller than the insertion penalty, since the flowpeak value is almost equally likely to be derived from a 1-mer compared to a 2-mer, but not as likely from a 3-mer.

### Insertion validation

As 454 data are generated from an underlying peak interpretation of sequential flows, some inserts are not likely to occur. For example, given the default 454 flow-order, “TACG”, and the flows; 3.05(T) and 2.95(C), thus a “TTTCCC”, an adenine (A) insert between the T- and C-peak are more likely compared to a guanine (G) as no G has been flowed between the peaks and the T- and C-peaks are strong evidence of the sequentiality of the T- and C-mer, with reservation for a false negative A-mer in between. This rule is applied in the alignment algorithm to avoid these types of unlikely inserts (the *-V* parameter). Furthermore, if 454 data is used the A-mer peak in between is examined to determine the insertion penalty. For example, if the flowpeak value is 0.49(A) the factor, *f*, as given by Equation 3 is *1*–*0.01/k*, and the resulting insertion penalty associated with an A-insert is low.

### Implementation

As the *m*_*n*_ function is not dependent on the protein sequence, a query profile where the value of the *m*_*n*_ function is pre-calculated can be constructed. Given such a query profile, the calculation cost of the cell-score is not much different from the regular Smith-Waterman-Gotoh algorithm cost, where just the values of four extra states are tested at each cell, see Equation 1. The algorithm is implemented, using C++, in a tool called HAXAT. HAXAT is available as open source and takes a wide range of parameters (see manual). HAXAT implements no heuristics and performs a complete traced alignment for all database/query combinations. HAXAT is available at http://www.bioinfo.ifm.liu.se/454tools/haxat.

## Competing interests

The authors declare that they have no competing interests.

## Supplementary Material

Additional file 1 **HAXAT parameter evaluation setup.** Name: haxat-parameter-evaluation.zip. Format: Zip compressed evaluation code. Title: HAXAT parameter evaluation setup. The file contains scripts for running the evaluation as well as all the binaries and results included in the manuscript. See the enclosed README for more information. Click here for file

Additional file 2 **Figure S1. **Double/Single gap penalty ratio for the non-454 aware model. The figure describes the alignment accuracy through Matthews Correlation Coefficient (y-axis) of the homopolymer insertion/deletion recovery versus the double/single gap penalty ratio (x-axis). The accuracy is given for each of the seven degrees of difficulty, i.e. alignments against targets of an identity ranging from 100% down to 40% identity. Furthermore, the mean accuracy for all seven degrees of difficulty, at each gap penalty, is also shown by squares and a dashed line. Click here for file

Additional file 3 **Figure S2. **Double/Single gap penalty ratio for the 454-aware model, without flowpeak information. The figure describes the alignment accuracy through Matthews Correlation Coefficient (y-axis) of the homopolymer insertion/deletion recovery versus the double/single gap penalty ratio (x-axis). The accuracy is given for each of the seven degrees of difficulty, i.e. alignments against targets of an identity ranging from 100% down to 40% identity. Furthermore, the mean accuracy for all seven degrees of difficulty, at each gap penalty, is also shown by squares and a dashed line. Click here for file

Additional file 4 **Figure S3. **Double/Single gap penalty ratio for the 454-aware model, using flowpeak information. The figure describes the alignment accuracy through Matthews Correlation Coefficient (y-axis) of the homopolymer insertion/deletion recovery versus the double/single gap penalty ratio (x-axis). The accuracy is given for each of the seven degrees of difficulty, i.e. alignments against targets of an identity ranging from 100% down to 40% identity. Furthermore, the mean accuracy for all seven degrees of difficulty, at each gap penalty, is also shown by squares and a dashed line. Click here for file
