# Supplementary figures and images for: Highly improved homopolymer aware nucleotide-protein alignments with 454 data
Source: BMC Bioinformatics. 2012 Sep 12;13:230. doi: 10.1186/1471-2105-13-230 (PMC3568017; doi:10.1186/1471-2105-13-230)

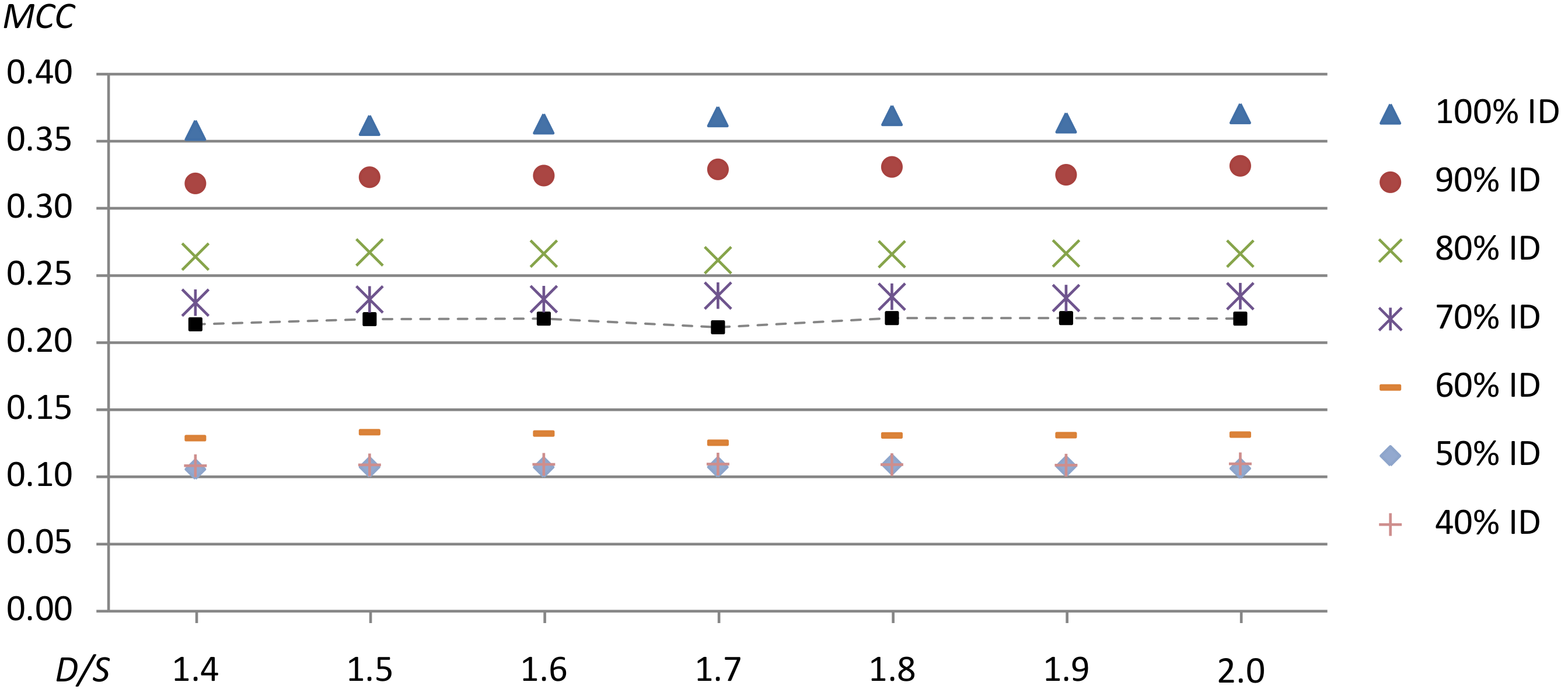

Supplement: Additional file 2 — Figure S1. Double/Single gap penalty ratio for the non-454 aware model. The figure describes the alignment accuracy through Matthews Correlation Coefficient (y-axis) of the homopolymer insertion/deletion recovery versus the double/single gap penalty ratio (x-axis). The accuracy is given for each of the seven degrees of difficulty, i.e. alignments against targets of an identity ranging from 100% down to 40% identity. Furthermore, the mean accuracy for all seven degrees of difficulty, at each gap penalty, is also shown by squares and a dashed line. [file 1471-2105-13-230-S2.tiff]

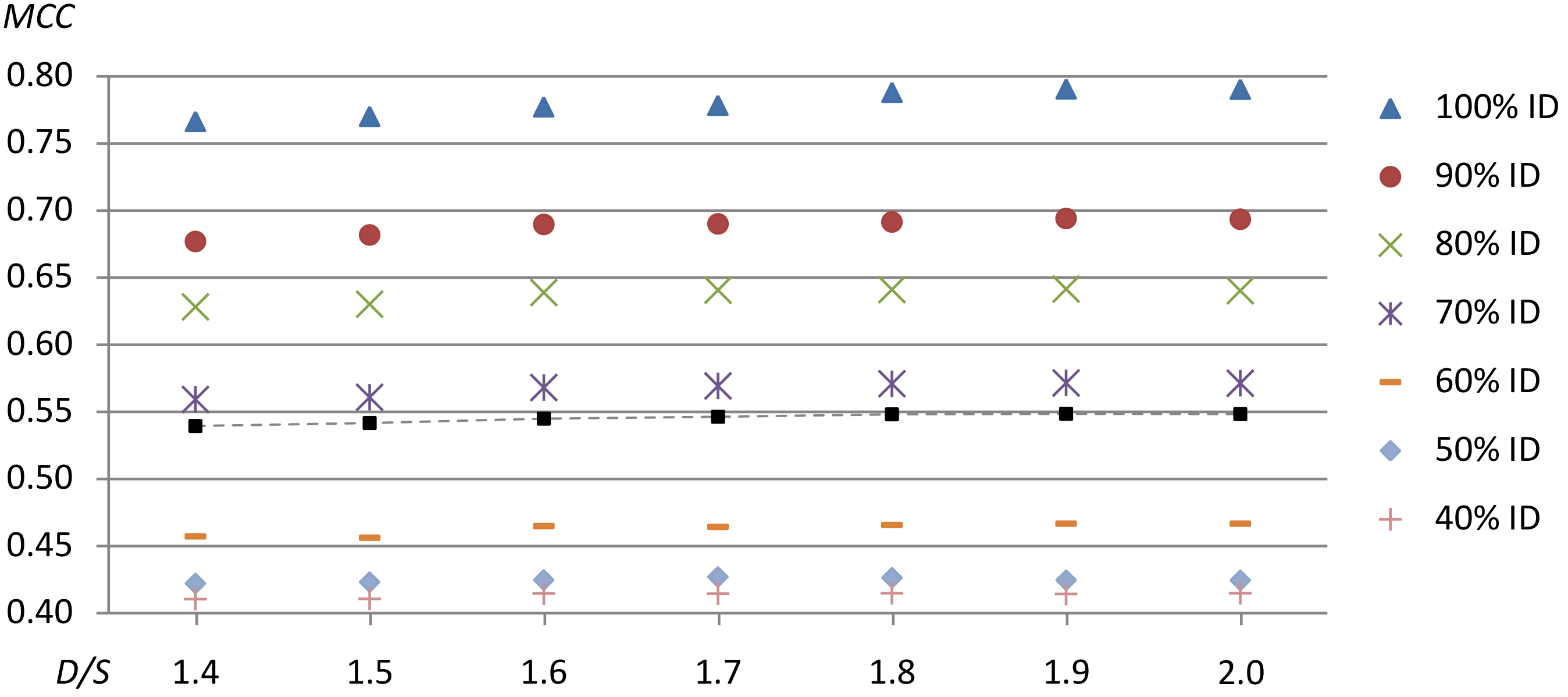

Supplement: Additional file 3 — Figure S2. Double/Single gap penalty ratio for the 454-aware model, without flowpeak information. The figure describes the alignment accuracy through Matthews Correlation Coefficient (y-axis) of the homopolymer insertion/deletion recovery versus the double/single gap penalty ratio (x-axis). The accuracy is given for each of the seven degrees of difficulty, i.e. alignments against targets of an identity ranging from 100% down to 40% identity. Furthermore, the mean accuracy for all seven degrees of difficulty, at each gap penalty, is also shown by squares and a dashed line. [file 1471-2105-13-230-S3.tiff]

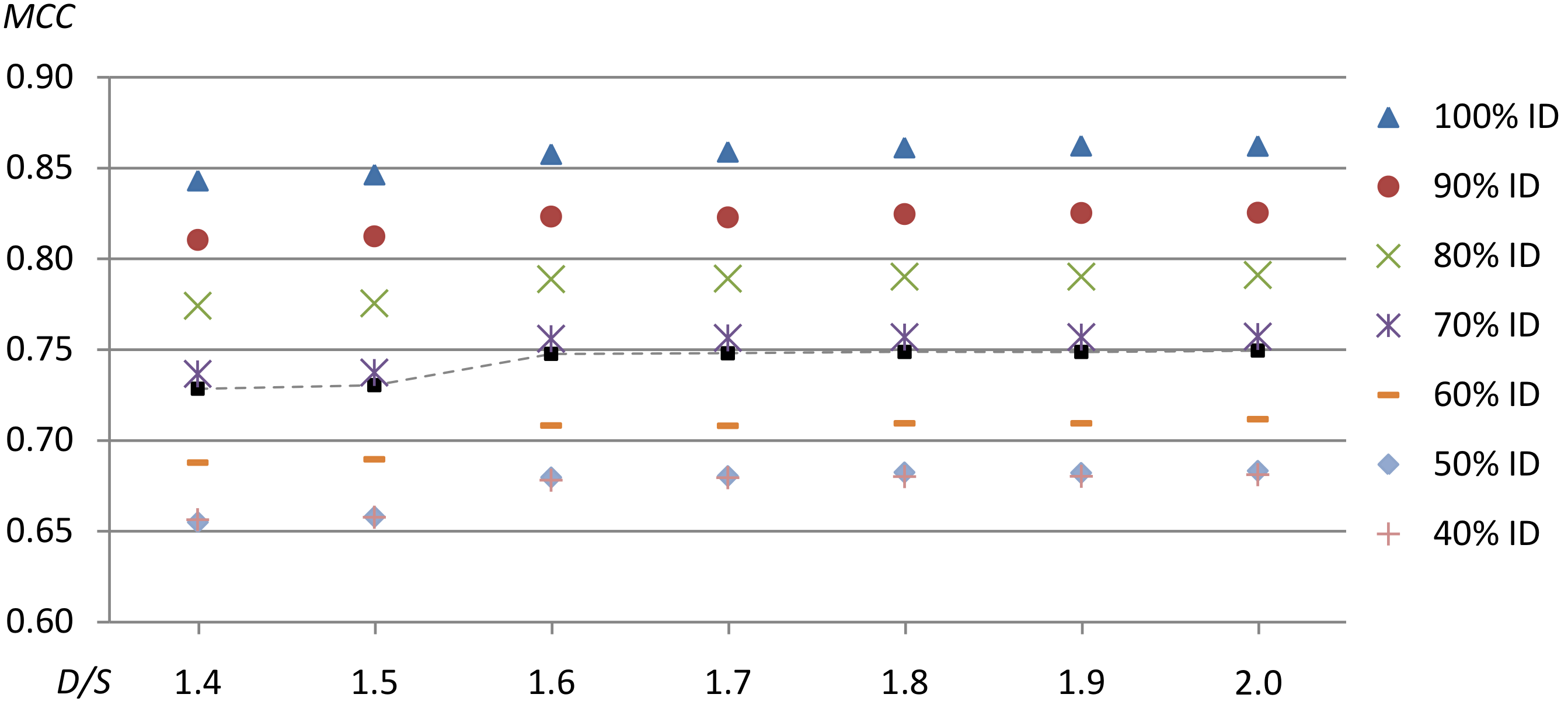

Supplement: Additional file 4 — Figure S3. Double/Single gap penalty ratio for the 454-aware model, using flowpeak information. The figure describes the alignment accuracy through Matthews Correlation Coefficient (y-axis) of the homopolymer insertion/deletion recovery versus the double/single gap penalty ratio (x-axis). The accuracy is given for each of the seven degrees of difficulty, i.e. alignments against targets of an identity ranging from 100% down to 40% identity. Furthermore, the mean accuracy for all seven degrees of difficulty, at each gap penalty, is also shown by squares and a dashed line. [file 1471-2105-13-230-S4.tiff]
